# Atraumatic Restorative Treatment compared to the Hall Technique for occluso-proximal cavities in primary molars: study protocol for a randomized controlled trial

**DOI:** 10.1186/s13063-016-1270-z

**Published:** 2016-03-31

**Authors:** Daniela Hesse, Mariana Pinheiro de Araujo, Isabel Cristina Olegário, Nicola Innes, Daniela Prócida Raggio, Clarissa Calil Bonifácio

**Affiliations:** Department of Cariology, Endodontics and Pedodontology, Academic Centre for Dentistry Amsterdam (ACTA), Gustav Mahlerlaan 3004, 1081 LA Amsterdam, The Netherlands; Orthodontics and Pediatric Dentistry Department, Dental School, University of São Paulo (USP), Av. Prof. Lineu Prestes, 2227, 05508-000 São Paulo, Brazil; School of Dentistry, University of Dundee, Nethergate, Dundee, DD1 4HN Scotland, UK

**Keywords:** Atraumatic restorative treatment (ART), Caries treatment, Clinical studies/trials, Glass ionomer cements, Pediatric dentistry, Primary teeth, The Hall Technique

## Abstract

**Background:**

In many parts of the world, school-age children have high dental treatment needs; however, there is often low, or no, dental care provision. Although Atraumatic Restorative Treatment (ART) was developed to address this, its survival rate in occluso-proximal lesions is low. An alternative, the Hall Technique (HT) has shown better relative outcomes for occluso-proximal lesions, but has not been directly compared to ART or tested in field settings. This trial will compare ART and the HT for the most clinically- and cost-effective strategy for managing occluso-proximal lesions in primary molars, in a school setting, using low-technology and child-friendly dental techniques.

**Methods/Design:**

This two-arm, parallel group, patient-randomized controlled, superiority trial will have treatment provided in schools. Schoolchildren (*n* = 124, age 6–8) with at least one occluso-proximal carious primary molar lesion will have random allocation to treatment with ART or HT. Baseline measures and outcome data will be assessed through participant report, clinical examination and parent report/questionnaires. The primary outcome is survival rate, a composite measure of absence of Minor Failures (a defect in the restoration/crown, but not interfering with tooth health) and Major Failures (signs or symptoms of irreversible pulp damage, such as dental fistula/abscess, tooth fracture or failures that cannot be repaired). Secondary outcomes are: (1) child-reported discomfort, (2) childrens’ and (3) parents’ concerns around dental appearance and (4) acceptability of treatments, (5) occlusal-vertical dimensions (OVD) changes, (6) plaque index, (7) gingival health, (8) decayed, missing, filled teeth in permanent teeth (DMFT)/decayed, missing, filled teeth in primary teeth (dmft), (9) oral health-related-quality of life, reported by children and parents/caregivers, (10) the incremental cost-effectiveness, and (11) operator effect. A trained and calibrated examiner will evaluate the treated teeth after 1 week, then 1, 6, 12, 24 and 36 months post treatment. Kaplan-Meier and Cox regression tests will be used to investigate the primary outcome. The Mann-Whitney or *t* test, Friedman test, paired *t* test or Wilcoxon test and Ordinal Logistic Regression Analysis will be used to analyze the secondary outcomes.

**Discussion:**

The results of this trial will support decision-making by clinicians and policy-makers for managing occluso-proximal lesions in settings with constrained resources and limited dental access.

**Trial registration:**

www.clinicaltrials.gov, NCT02569047, registered 5 October 2015.

**Electronic supplementary material:**

The online version of this article (doi:10.1186/s13063-016-1270-z) contains supplementary material, which is available to authorized users.

## Background

The occurrence of cavitated caries lesions is still a problem in developed and developing countries [1], with an increasing prevalence, particularly in developing communities, conflicting with a general descending trend in prevalence worldwide [2]. In this context, the Atraumatic Restorative Treatment (ART) was developed. ART is a two-part strategy for the management of caries: the restorative step, and the essential adjunctive educational-preventive strategies [3]. The performance of ART restorations for longevity has been evaluated through clinical trials with the results of numerous studies showing that ART performs well for occlusal cavities in primary and permanent teeth [4–6] but there appear to be much lower success rates for occluso-proximal ART restorations in permanent and primary teeth [3, 4, 7–9]. Thus, occluso-proximal cavitations are now the main focus for contemporary research [10]. For primary teeth, reported restoration success rates range from 50 to 75 % in the first 2 years [11-14] with reported survival rates after 3 years of evaluation even lower [9], dropping to only 20 % in some cases [15].

Recently, the paradigm around the ideal management of carious lesions has been changing. Conventional restorative approaches, with its emphasis on the complete removal of carious tooth tissue followed by placement of a restoration [16, 17] has been substituted by more biological and less invasive approaches, focusing on biofilm control and disruption of the cariogenic biofilm environment to arrest caries [18–20]. The Hall Technique (HT) fits this philosophy. A preformed metal crown (PMC) is cemented, using glass ionomer cement (GIC), over the carious tooth, without tooth preparation or caries removal. This seals the cariogenic biofilm under the crown [21]. Studies have reported high survival rates of HT, with results of 98 % success rate after 1 year of evaluation [22] and 95 % after 23 [21] and 48 months [23].

Although HT is recommended for the management of dental caries in primary molars involving two or more surfaces and evidence of its efficacy had been published [21–23], these have all been carried out in clinical dental settings and have not been compared to ART. There are no clinical trials of the HT in a field setting and no direct comparisons of the longevity of occluso-proximal ART restorations with HT.

Therefore, the aim of this study is to evaluate the survival rate of occluso-proximal ART restorations compared with the HT in primary molars. Furthermore, self-reported patient discomfort, perception and concerns related to dental appearance, acceptance in relation to treatments reported by children and their parents/caregivers, evaluation of occlusal-vertical dimension (OVD), evaluation of cost-effectiveness, the impact of the two techniques on the oral health-related quality of life (OHRQoL) and the influence of the operator experience on the survival rate of treatments will be investigated.

## Methods

### Ethical considerations and registrations

This protocol has been approved by the Research Ethics Committee of Dental School, University of São Paulo (protocol 1.293.935). The study protocol has been registered on ClinicalTrials.gov (NCT02569047 – 5 October 2015) and written following Consolidated Standards of Reporting Trials (CONSORT) guidelines for randomized trials of non-pharmacologic treatment [24] and the Standard Protocol Items: Recommendations for Interventional Trials guidelines for clinical trial protocols (SPIRIT - http://www.spirit-statement.org/spirit-statement/).

The study will be conducted in Tietê, a city in the state of São Paulo, Brazil. Informed consent will be obtained from children’s parents or guardians before participation in the study. Each child must also assent to participate. Participants’ confidentiality will be ensured using identification code numbers and the information recorded will be available only to researchers.

### Study design

**Fig. 1:**
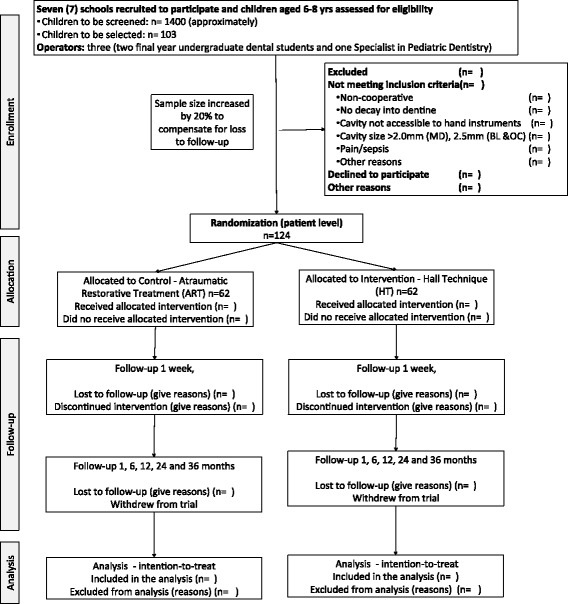
Consolidated Standards of Reporting Trials (CONSORT) flow diagram of patient randomization

This is a two-arm, parallel group, patient-randomized controlled, superiority trial with treatment provided in a school setting. The participants will be allocated to one of the two arms in order to compare different options for arresting occluso-proximal caries lesions (Fig. 1).

### Sample size calculation

The sample size was calculated based on the primary outcome – treatment survival, using the log-rank test in survival analysis. This involved a two-tailed test based on survival rate reported for ART (62 %) [13] after 2 years of follow- up, using the absolute difference of 25 % between groups, *α* of 5 % and power (strength) of 80 %. This gave an estimate of 103 children (with one tooth each treated within the study) to be recruited. After increasing by 20 % to compensate for participant loss to follow-up, the final sample size was set at 124 children (and 124 teeth).

### Participant selection

Children (6–8 years old) will be screened at seven primary schools and 124 who meet the inclusion criteria will be selected. Only the children whose parents or legal guardians signed the informed consent form and who assent to be part of the study will be included in the research. Children will be evaluated at the school in empty classrooms, prepared for the oral-examination, and receive instructions on oral health, particularly in relation to oral hygiene/toothbrushing and sugar consumption.

All phases of this trial will be carried out in the school where the child studies. First, the operators will carry out an intra-oral examination using a dental mirror, cotton rolls and periodontal probe. All information will be recorded in individual forms (see Additional file 1). The biofilm will be removed using gauze. The criteria for caries presence will be those of the World Health Organization (WHO) [25] and the decayed, missing, filled teeth in permanent teeth (DMFT) and decayed, missing, filled teeth in primary teeth (dmft) will be recorded [26].

### Participant inclusion criteria

The inclusion criteria for participants are: children whose parents or legal guardians accept and sign the consent form; children who assent to participation; aged between 6 and 8 years; exhibiting generally cooperative behavior; with good general health conditions; presenting at least one occluso-proximal lesion in a primary molar.

### Tooth inclusion criteria

Radiographic diagnostic facilities are not available in this setting, so only clinical diagnosis will be used. Specifically, the caries lesions inclusion criteria are: caries limited to the occluso-proximal surfaces and extending to dentine, accessible to hand instruments used in ART, absence of pain, fistula or abscess near the selected tooth; absence of pulp exposure; absence of pathological mobility; and cavity size smaller than 2.0 mm mesio-distally and 2.5 in the occluso-cervical and bucco-lingual directions measured with a WHO-graded periodontal probe.

In cases where the child has more than one occluso-proximal cavity eligible for inclusion in the study, only one cavity will be selected. In those cases, the child will have all teeth that meet the inclusion criteria numbered. Those numbers will be written on pieces of paper, folded and placed in an opaque envelope. An independent dentist who is not involved in the research will be responsible for selecting one of the papers, containing the tooth number that will be included in the research. Dentists from the municipality where the trial will be carried out, the operators of this trial and new groups of final year undergraduate dental students will perform all other necessary treatments. If necessary, the participants will continue to receive dental treatments in the municipality where the investigation will be carried out after completing this trial.

### Random allocation

The selected children will be assigned, by random allocation, to have their tooth treated with ART (control group) or the HT (experimental group). The randomization sequence will be generated electronically (http://randomization.com/) and to ensure allocation concealment, the randomly generated sequence will be sealed in opaque envelopes. An independent dentist from the municipality where the trial will be carried out will designate the allocation of each child using the opaque envelopes. Therefore, the envelope will be opened during the treatment, but only after the child is ready to receive treatment.

### Operators

Operators will be two final-year undergraduate dental students and one specialist in pediatric dentistry with 3 years of experience treating children. They were trained in both treatments. Training comprised a lecture on ART and the HT by clinicians experienced in the treatments and experienced in clinical trials. Also, the undergraduate students participated in a hands-on laboratory-based workshop for the handling and application of the materials and practicing carrying out both ART and the HT by clinicians experienced in the treatments. All operators will also undergo 2 weeks of training with children, using the different techniques before starting the study. This phase will be carried out in the field setting with similar children to those participating in the study, under the direct supervision of clinicians experienced in ART and HT.

Each child will be allocated to be treated by one of the operators with the aid of the random allocation list. All treatments will be performed on the school premises, in field conditions without the use of dental chair or other facilities from a clinical environment.

### Protocols for interventions

#### Control arm – Atraumatic Restorative Treatment (ART)

The teeth will be prepared according to the ART approach proposed by Frencken et al. [3], and no local anesthesia will be used as per standard ART protocol. The cavities will be filled according to Yu et al. [14] (Table 1).

**Table 1:** Protocol for Atraumatic Restorative Treatment (ART) restorations: preparing the teeth according to Frencken et al. [3] and restoration of cavities according to Yu et al. [14]

1. Preparing the cavity • Place cotton wool rolls alongside the tooth to be treated. • Remove plaque from tooth surface with wet cotton wool pellets. • Dry the tooth surface with dry cotton wool pellets. • If necessary, make the entrance of the cavity wider with a dental hatchet. • Remove the carious dentine with excavators, until the enamel-dentine junction is caries free. • Fracture off unsupported thin enamel with the hatchet. • Clean the cavity with wet and then dry with cotton wool pellets. • Remove the caries near the pulp carefully. In this area only the completely soft and demineralized tissue must be removed. • Clean the cavity again with wet cotton wool pellets. • Complete the procedure by drying the cavity with dry cotton wool pellets. • Place a matrix strip between the teeth. This matrix must be pre-curved. Insert a wedge to support the strip under the contact point at the gum margin. Advise the child that he/she might feel a little uncomfortable during this procedure. 2*.* Conditioning the cavity • Apply one drop of GC Cavity Conditioner liquid (GC Europe, Leuven, Belgium) on paper pad. • Dip a cotton wool pellet in clean water. • Remove excess of water from the cotton wool pellet by lightly touching against a dry cotton wool roll, tissue or gauze. • Dip the moist cotton wool pellet in the GC Cavity Conditioner liquid. • Condition the cavity and adjacent fissures with the liquid for 10–15 seconds. • Wash the cavity and fissures immediately with three sequences of cotton wool pellets, dipped in clean water. • Dry the cavity with three sequences of dry cotton wool pellets. 3. Restoring the cavity • Ensure that the tooth is kept dry during the restoration phase • The capsule of EQUIA Forte, capsules (GC Europe, Leuven, Belgium) should be activated and mixed. Before activation, shake the capsule or tap its side on a hard surface to loosen the powder. To activate the capsule, push the plunger until it is flush with the main body. Immediately place the capsule into a metal capsule applier and click the lever once and it will be activated. Immediately remove the capsule and set it into a mixer (or an amalgamator) and mix for 10 seconds • Remove the capsule from mixer and place into a metal capsule applier again. Make two clicks to prime the capsule. Within 10 seconds maximum after mixing, start to extrude the mixture directly into the preparation. Extra care should be taken to avoid moisture contamination or drying out • Place the index finger on the restorative material, press and remove finger sideways after a few seconds • Remove visible excess of glass ionomer cement (GIC) with a medium or large excavator • Wait 1–2 minutes till the material feels hard, whilst keeping the tooth dry • Remove matrix turning this to the other side (adjacent tooth) and wedge carefully and check the bite using articulation paper and adjust the height of the restoration with the applier/carver if needed • Using a micro-tip applicator, apply G-Coat Plus (GC Europe, Leuven, Belgium) to the occlusal and proximal surface of restoration. A new metallic matrix band and wedge must be applied and light cured for 20 seconds at occlusal, 20 seconds at buccal and 20 seconds at the lingual surface. G-Coat Plus is indicated to seal and protect the surface of glass ionomer • Remove the matrix, wedge and cotton wool rolls • Child will be told not to eat for at least 1 hour after the treatment. A sticker with the time at which they are allowed to eat again will be pasted on their t-shirt	

#### Intervention Arm – Hall Technique (HT)

The treatments will be carried out according to the HT protocol of Innes et al. [27]. No local anesthesia will be used as it is not required (no dentine is removed) and as per standard HT (Table 2).

**Table 2:** Protocol for carrying out Hall Technique restorations, following the Innes et al. [27] protocol

First visit: • Assess the tooth shape, contact points/areas and the occlusion. • Use orthodontic separators to create space for fitting a Hall crown, unless there are no contact points. In order to protect the airway, the child will be sat upright • Thread two lengths of dental floss through the separator. Stretch the separator and floss taught and floss through the contact point briskly and firmly until the leading edge only is felt “popping through” the contact point. Remove the floss and make a second appointment with the patient 3 to 5 days later. Second visit: • Remove the separator with an excavator. • Gently remove loose plaque and food debris only from the cavity. • Assess the occlusion: measure the patient’s occlusal-vertical dimensions (OVD) with a millimeter probe using the distance between the most coronal points of the primary canines in order to assess the degree of overbite after mounting of the crown. • Protect the airway by placing a gauze swab square between the tongue and the tooth to be crowned. • Select the correct crown size (Stainless Steel Crowns, 3 M™ ESPE™, St. Paul, MN, USA). The crown should covers all the cusps and approaches the contact points, with a slight feeling of “spring back.” You should aim to fit the smallest size of crown which will seat. • Keep the treatment area free from saliva by isolating the tooth with cotton wool rolls. • Dry the inside of the crown with dry cotton pellets. • Mix the encapsulated glass ionomer cement (GIC) (Fuji I, GC Europe, Leuven, Belgium) for 10 seconds, according to the manufacturer’s instructions. • Load the crown generously with GIC (at least two thirds full). Avoid air blows and voids. • Place the crown over the tooth and seat the crown into place by finger pressure or ask the child to bite it into place. • Check the crown position as soon the crown is fitted. • Wipe away the excess GIC with a cotton wool roll or the gauze swab used to protect the airway. • Place a cotton wool roll between the crown and the opposing tooth and ask the child to bite firmly on the crown for another 2–3 minutes. • Remove excess cement, flossing between the contacts. • Blanching usually disappears within minutes. The occlusal discrepancy should resolve in a few weeks. • Measure the degree of bite opening and record in the notes. If excessive, then consider removing the entire crown. • Check the buccal relationship of the crowned tooth with its opposing number. If there is a displacing contact, resulting in a cross bite, then manage as for excessive bite propping.	

### Data analyses

Outcome data for the trial will be analyzed as intention-to-treat (ITT) so that all participants randomized and all events will be accounted for in the primary analysis. However, if (1) more than 10 % of children cannot tolerate either one or both of the treatments at the time of treatment, or (2) there is a difference greater than 20% in the dropouts between the arms (therefore raising the index of suspicion that there is a problem with tolerance of the treatments or other problem with delivery), we will also carry out an “on treatment” (also known as per-protocol) analysis and interpret the differences between ITT and “on treatment” appropriately.

### Primary outcome evaluation

One trained and calibrated examiner will carry out the follow-up evaluations. The examiner will undergo a training period according to the criteria adopted and in order to calculate intra-observer reliability, 20 % of the sample evaluated at the first evaluation period will be re-evaluated after 2 weeks. The intra agreement will be calculated and score above 0.7 will be accepted.

The treatments will be classified as “success” when they present as clinically satisfactory (that is where there have been no failures). Failures will be scored as “Minor Failures” and “Major Failures” (adapted from Innes et al., [21]). The Minor Failures will be those in which there is a defect in the restoration/crown, but it does not interfere with the tooth health. The Major Failures are when there are signs or symptoms of irreversible pulp damage, such as dental fistula/abscess, tooth fracture or failures that cannot be repaired (Table 3).

**Table 3:** Evaluation criteria for the assessment of treatments (modified from Innes et al. [21])

Success	• satisfactory restoration/crown, no intervention required • no clinical signs or symptoms of pulp pathology • tooth exfoliated
Minor Failures	• secondary caries, or new lesions detected clinically • crown presents perforation • restoration fracture or wear - intervention required • loss of restoration - tooth that can be re-restored • crown loss - tooth able to be re-treated • reversible pulpitis, which could be treated without the need for extraction or pulpotomy
Major Failures	• irreversible pulpitis or dental fistula/abscess, requiring pulpotomy or extraction • loss of restoration/crown - tooth does not capable of being re-restored

The primary outcome of survival rate will be analyzed primarily as a composite of Minor and Major Failures but we will also conduct a further analysis by dividing the outcomes into two levels: assessment of treatment survival (Minor Failure) and tooth survival (Major Failure).

#### Treatment (restoration) survival

ART restorations and HT scored as satisfactory will be considered “successful,” while those presented Minor and/or Major Failures will be considered as “failed.”

#### Tooth survival

Treatments will be considered as being “successful” for teeth where treatments are scored as satisfactory or where there has been Minor Failures. Teeth that present Major Failures will be considered as “failure for tooth.”

### Secondary outcomes evaluation

For the assessment of discomfort, the Wong-Baker FACES pain rating scale, an ordinal six-point scale ranging from 0 to 5, will be used [28]. A score of 0 shows a smiling face, indicating no discomfort, whereas a score of 5 shows a crying and sad face, indicating great discomfort. This method has been validated for the assessment of pain and discomfort in children, before and after treatments [28, 29].

To evaluate the perception and concerns related to dental appearance, the Child’s and Parent’s Questionnaire about Teeth Appearance [30] will be used and applied through an interview with children in the school, as well as being filled out by their parents/caregivers at home. This instrument has a version for children and a version for their parents, including questions related to physical, psychological and social order, beyond the perceptions of color change and other esthetic conditions related to the child’s teeth. The questionnaire has five questions with the first three items assessing how the child felt uncomfortable (physical domain of the concept of health), concerned (psychological domain) and avoided smiling (social field) due to the appearance of his/her teeth. An additional item with four sub-items assesses the perception of children and their parents/caregivers about the appearance, color and health of teeth and the last item assesses the opinion of children and their parents/caregivers regarding the satisfaction with the teeth color.

The questionnaire for the evaluation of acceptance in relation to treatment carried out will be applied through interview with children in the school as well as being filled out by their parents/caregivers at home. These questionnaires are based on questionnaires used by Bell et al. [31]. They were rewritten in order to be useful for both the ART and the HT (see Additional file 2). A dentist who is fluent in both languages translated the questionnaires from English to Portuguese. The questionnaire for the children contains six items and employs a five-point pictorial Likert scale. The possible responses are: strongly agree, agree, indifferent, disagree and strongly disagree. The parental questionnaire has five questions with the same possible responses.

**Fig. 2:**
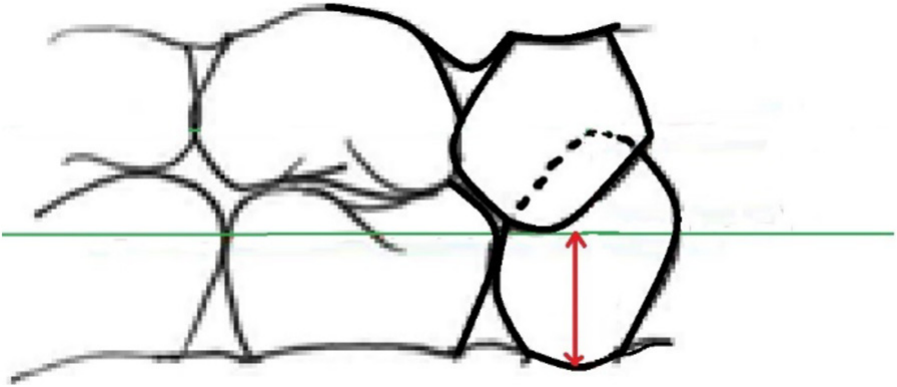
Method for measuring occlusal-vertical dimension (OVD) using a probe with markings at 1, 3, 6, and 9 mm

The assessment of the OVD will be performed according to a modified version of van der Zee and van Amerongen [32]. Ideally, this will be measured using the canines on the same side of the jaw as that where the treatment takes place. If this is not possible we will measure the OVD on the contralateral side of the jaw. If there are no canines present, we will measure the OVD using the first primary molars. The OVD measurement and the teeth measured will be recorded. One examiner will measure the OVD before and after the treatment, and in the subsequent evaluations. The examiner will be trained and calibrated according to the protocol adopted for this measurement. The OVD will be measured using a probe with markings at 1, 3, 6 and 9 mm. The distance from the lowest point of the gingiva, around the lower canine on the vestibular side up to the point where the tip of the upper canine ends, will be measured. This tip does not have to be directly above the lowest point in the gingiva around the lower canine as an imaginary line will be being drawn through the tip of the antagonist and the distance measured from the gingiva to the line (Fig. 2).

Plaque [33] and gingival bleeding indices [34] will be recorded on the treated tooth as well as obtained by averaging the measurements from seven index teeth [22] (Table 4).

**Table 4:** Evaluation criteria for the assessment of plaque index [33] and gingival health [34]

Plaque index scores [33]	• 0: no plaque • 1: thin visible plaque, difficult to identify • 2: thick visible plaque, easily detected.
Gingival health scores [34]	• 0: Normal: the gingival tissue appears firm, with pinkish or pale pink and opaque surface, with thin margins and variable degree of stippling. Contact with millimeter probe show its firmness • 1: slight inflammation: the gingival margin has slight change of color (reddish or bluish red) and is slightly swollen. Does not bleed after gentle probing • 2: moderate inflammation: the gingival tissue has become swollen (rounded edge, or bright red/blue). There is bleeding after gentle probing; 3: severe inflammation: the gingival tissue has markedly red or bluish red, swollen and enlarged, with ulcerations. Tendency to spontaneous bleeding

For the assessment of changes in DMFT/dmft, the same criteria for the evaluation of caries presence used at the baseline evaluation will be used [25] and the DMFT and dmft will be recorded [26].

An incremental cost-effectiveness ratio (ICER) will be calculated. The average cost per treatment will be quantified for both ART and the HT. Effectiveness will be measured by percentage of treatment survival. A microcosting will be carried out to estimate the direct costs of treating children with ART or the HT. This will include the capital costs of all equipment and instruments, materials and overheads, and the costs of time and labor. Additionally, the procedures will be timed using a stopwatch, started when the child has his/her mouth open and the operator is about to start the restorative intervention and stopped when the child stands up from the treatment table. The initial cost of treatments will be calculated, taking into account the parameters described in the Table 5. Therefore, the initial costs of treatments will be calculated by summing the expenses of capital cost, material cost and labor cost [35]. The ICER will be generated per treatment group by dividing the average initial cost by the survival after 3 years:

**Table 5:** Parameters used to calculate the costs per treatment

Capital cost	• Fixed cost of equipment and instruments such as the cost of autoclave and examination kits. For analysis we will assume that the lifespan of a dental instrument is approximately 3 years or 1095 days (constant depreciation rate).
Materials cost	• supplies such as gloves, masks, articulating paper, restorative material and PMCs. Their accumulated costs will be estimated per restoration.
Labor costs	• salaries of dentists and dental nurses delivering treatment will be calculated using the top point in their Brazilian Public health Service salary scales for the city in which the treatment is being provided. The labor cost per day will be divided by the number of restorations/ PMCs placed per day.

$$ ICER=\left({\mathrm{cost}}_i\hbox{-} {\mathrm{effectiveness}}_i\right)/\left({\mathrm{cost}}_c\hbox{-} {\mathrm{effectiveness}}_c\right)\;\mathrm{where}\;i=\mathrm{intervention}\;\mathrm{and}\;c=\mathrm{control}. $$

The perceptions of parents and children regarding the OHRQoL will be evaluated using the Child Perceptions Questionnaire (CPQ). This questionnaire takes into account the cognitive abilities and lifestyles, and is validated for Brazilian children aged from 8 to 10 years (CPQ_8–10_) [36, 37]. The instrument consists of 29 questions, distributed into four domains (child symptoms, function, psychological, and self-image/social interaction domains) and in a family impact section (oral symptoms, functional limitations, emotional well-being and social well-being). Answers will be recorded through a Likert scale from 0 to 4: 0 = never, 1 = once or twice, 2 = sometimes, 3 = often, 4 = very often. The minimum possible score is 0, the maximum possible score is 116, with higher scores indicating a greater negative impact on OHRQoL. The CPQ_8–10_ questionnaires will be applied as an interview to children in the school. Furthermore, the parents will also be invited to answer the Brazilian version of the Parental-Caregiver Perceptions Questionnaire (P-CPQ) at home [38]. The questionnaire is composed of 35 questions that assess the perceptions of parents and/or guardians regarding the impact of oral diseases on the quality of life of children aged 6 to 14 years as well as a rating scale consisting of 14 questions that assess the effects of oral disorders in family functioning. Questions 1 and 2 refer to the overall perception of those responsible for oral health and the child’s overall well-being. Answers will be recorded using a Likert scale from 0 to 4: 0 = excellent, 1 = very good, 2 = good, 3 = fair, 4 = bad. The remaining questions are divided into four broad categories: oral symptoms (questions 3–8), functional limitations (questions 9–16), emotional well-being (issues 17–24) and social welfare (questions 25–35). Questions 36–49 refer to the impacts of oral disorders in family welfare. Response options range from 0 to 5: 0 = never, 1 = once or twice, 2 = sometimes, 3 = often, 4 = every day or almost every day; 5 = do not know. The total score is obtained by summing the scores of all issues. Higher scores indicate greater the impact of oral diseases on the quality of life.

### Follow-up examinations

At baseline, the outcome, child discomfort associated with treatment will be recorded:

- ART group: before and immediately after treatment
- HT group (on two occasions): 1 – before and immediately after the placement of a separator when it is used, and 2 – before and immediately after the placement of the PMC

Perceptions and concerns related to dental appearance will be measured before and after treatment – for children and parents.

Questionnaires assessing acceptance of treatment will be given after treatment – for children and parents.

OVD measurement will be carried out before and after treatment.

Plaque index, gingival health, DMFT/dmft scores (before treatment) and the OHRQoL questionnaires will be recorded before treatment – for children and the OHRQoL questionnaire will be given to parents before treatment. Children will be assessed after 1 week, and then after 1, 6, 12, 24 and 36 months. At 1-week follow-up, the outcomes related to treatment evaluation, OVD measurement, plaque index and gingival health will be re-evaluated. At 1 month follow-up, the outcomes related to treatment evaluation, OVD measurement, plaque index and gingival health, DMFT/dmft scores and the questionnaire OHRQoL, will be re-evaluated. At other follow-ups (6, 12, 24 and 36 months), the outcomes related to treatment evaluation, OVD measurement, plaque index and gingival health, DMFT/dmft scores will be re-assessed (Table 6).

**Table 6:** Sequence of procedures performed for each recruited participants

		ART group								HT group							
		Baseline		Evaluation						Baseline		Evaluation					
Event	Completed by	Baseline examination appointment	Treatment appointment	1 week	1 month	6 months	12 months	24 months	36 months	Baseline examination appointment	Treatment appointment	1 week	1 month	6 months	12 months	24 months	36 months
Consent/Assent	Dentist/Dental student	X								X							
DMFT/dmft	Dentist/Dental student	X			X	X	X	X	X	X			X	X	X	X	X
Plaque index	Dentist/Dental student	X		X	X	X	X	X	X	X		X	X	X	X	X	X
Gingival health	Dentist/Dental student	X		X	X	X	X	X	X	X		X	X	X	X	X	X
Instructions of oral hygiene/diet	Dentist/Dental student	X								X							
Orthodontic separators	Dentist/Dental student									X							
Treatment	Dentist/Dental student		X								X						
Wong-Baker faces scale (pre/post treatment)	Child		X							X	X						
Questionnaire about teeth appearance (pre/post treatment)	Child/Parent		X			X					X			X			
Questionnaire about acceptance in relation to treatment	Child/Parent		X								X						
Measurement of OVD	Dentist/Dental student		X*	X	X	X	X	X	X		X*	X	X	X	X	X	X
Perceptions regarding OHRQoL	Child/Parent		X		X						X		X				
Treatment evaluation	Dentist/Dental student			X	X	X	X	X	X				X	X	X	X	X

*OVD will be measured before and immediately after treatment during the treatment appointment in Atraumatic Restorative Treatment (ART) and Hall Technique (HT) groups

*DMFT/dmft* decayed, missing, filled teeth in permanent teeth/decayed, missing, filled teeth in primary teeth, *OHRQoL* oral health-related quality of life, *OVD* occlusal-vertical dimension

### Data management

All collected data will be entered directly into predetermined files. Checking missing data, out of range values, illogical and invalid responses, will ensure data quality.

### Statistical analysis

The inter- and intra-examiner reproducibility for assessing the primary outcome and secondary outcome OVD measurements will be calculated using a weighted Kappa test.

#### Primary outcome

To verify the survival rate of treatments, as well as to evaluate the survival rate of the teeth examined, we will use a Kaplan-Meier survival analysis and the log-rank test. To evaluate the association between the outcome and patient variables we will use Cox regression test. The significance level for all analyses will be *p* <0.05.

#### Secondary outcomes

The Kolmogorov-Smirnov and Levene tests will be used to consider the normality distribution and homoscedasticity of the data, respectively. Depending on the data distribution, the Mann-Whitney test or *t* test will be applied to compare the degrees of discomfort, perception and concerns related to dental appearance reported by the child and their parents/caregivers; the child and their parent/caregivers’ reported acceptance of treatments among the two groups of intervention, as well as to evaluate the difference in mean scores of CPQ and P-CPQ among the two groups of intervention. The differences between the mean scores of the responses from the CPQ and P-CPQ questionnaire obtained at 1 month and at baseline will be calculated and tested, using a paired *t* test. The Friedman test will be used to compare the changes with regards to concerns related to dental appearance reported by the child and their parents/caregivers, changes in OVD; plaque index and gingival health. To evaluate the association between the secondary outcomes and children variables Ordinal Logistic Regression Analysis will be used. For cost-effectiveness analysis, the primary measure will be the survival rate of treatments. Therefore, as previously mentioned, the ICER will be generated per treatment group by dividing the average initial cost by the survival after 3 years:

$$ ICER=\left({\mathrm{cost}}_i-{\mathrm{effectiveness}}_i\right)/\left({\mathrm{cost}}_c-{\mathrm{effectiveness}}_c\right)\mathrm{where}\;i=\mathrm{intervention}\ \mathrm{and}\;c=\mathrm{control}. $$

The significance level for all analyses will be *p* <0.05.

### Data monitoring

The adverse events related to the treatments investigated in this trial are those related to dental treatment. So there is no Data Monitoring Committee, and independent oversight of trial data collection, management and analysis is undertaken by DPR. The chief investigator (DPR) has overall responsibility for the study and is custodian of the data.

### Harms

As previously stated, the adverse events related to the treatments investigated in this trial are those related to dental treatment. These effects are usually expected in any conventional dental treatment performed in pediatric dentistry clinical practice.

### Auditing

The collected data will be subject to audit by the coordinator and data queries raised if necessary.

### Dissemination policy

Results will be reported through peer-reviewed journals and by the municipality website. The results will be reported regardless of findings.

## Discussion

This trial aims to investigate which minimally invasive treatment applicable outwith the dental surgery setting, the HT or ART, has the best survival rate for occluso-proximal cavitated lesions in primary molars when applied in a low-technology setting. In addition, we aim to answer the question: how do these treatments compare for child self-reported discomfort associated with treatment, pain and infection rates, OVD changes, incremental cost-effectiveness, DMFT, DMFT/dmft changes, OHRQoL, perceptions, concerns and acceptance related to dental appearance reported by children and their parent/caregivers?

ART was developed approximately 30 years ago and involves the use of manual instruments to prepare cavities, followed by placement of a high-viscosity GIC [3]. This strategy for the management of caries has been tested in many settings with good outcomes for single surfaces [5, 6, 39]. However, the survival rate of ART for occluso-proximal lesions is low [13, 14]. As a result of this reduced longevity, there is some resistance within the dental and scientific community to recommending this technique for everyday use [40].

A recent systematic review of the literature has shown the longevity of occluso-proximal ART restorations in primary teeth to be similar that of conventional restorations using amalgam, composite resin and compomer, suggesting that the real problem might be related to the type/extent of cavity and not the restorative material [10]. However, preformed metal crowns offer physical protection to teeth affected by caries, through complete tooth coverage, as well as arresting caries progression [41]. In direct comparisons, their longevity has been found to be equal to or superior to restorations [42, 43]. A less invasive alternative method for using preformed metal crowns is the HT, where crowns are pushed over teeth with no tooth preparation or caries removal [27]. The HT has also been shown to have good longevity. According to Santamaria et al. [22], the success rate for the HT was 98 % after 1 year and showed superior efficacy against a non-restorative approach and a conventional restoration. Moreover, the HT has shown more favorable results for pulpal health and tooth longevity [21, 23]. However, the question regarding the superior performance of the HT and ART remains unanswered. This trial will test the HT and ART in field conditions, without dental clinic facilities. This could provide a possible solution to caries treatment for more extensive lesions in public health systems in developing countries.

The preferences of children, their parents/caregivers for one of the treatments is also being tested. Neither ART nor HT require local anesthesia and both have been found to be preferred to conventional restorative methods, by children, their parents/caregivers and dentists, and usable by inexperienced practitioners [21, 44–47]. Although both treatments, ART and HT, have shown to be well-accepted by children and parents, there is no research directly comparing the two techniques for this outcome. However, esthetics related to dental treatment can be a concern of parents and caregivers and there may be a difference in the perceptions of metal crowns compared to white GIC. Previous reports have noted that one of the reasons given by dental practitioners for not fitting stainless steel crowns for multi-surface lesions, extensive caries and those where pulpal treatment was performed, was because metal crowns are not cosmetically acceptable to the child or the parent, although most dentists did recognize crowns as the most durable restoration for primary molars [48, 49]. Threlfall et al. [48] also noted that although some parents had complaints regarding the crowns’ esthetics, once the dentist explain all the advantages, they agreed with the treatment. As no trials have been conducted to evaluate the perception and concerns related to dental appearance of ART and the HT, this trial will also investigate whether the appearance of the metal crown is a barrier to providing this treatment.

Because there is no tooth preparation or caries removal, the OVD tends to be increased after placement of a crown using the HT [21, 32, 50]. Although van der Zee and van Amerongen [32] reported that the occlusion re-establishes after 15 to 30 days, according to the authors, a major limitation in their study was the very small study population size at the 30-day evaluation (*n* = 8). Through frequent evaluation and more comprehensive capture of the study population in the school setting, this trial will test whether the occlusion re-establishes after crown cementation.

In order to define public health care policies, costs of treatment have to be taken into consideration. Cost-effectiveness is defined as the analysis of costs of alternative treatments to be offered to a population [51]. Cost-effectiveness analyses have been used to appraise ART sealants and restorations in children and elderly populations [35]. The HT has been subjected to clinical evaluation in several studies [21–23, 46, 50], and one recent investigation addressed the results regarding the cost-effectiveness of three strategies for treating carious primary molars [52]. However, that study was a modeling design research and the economic evaluation of HT used in children has not yet been evaluated in a clinical trial.

In our investigation we aim to carry out an incremental cost-effectiveness evaluation where the initial costs of treatments (the costs to produce 1 unit of ART restoration or HT-crown) will be combined with the survival rates obtained by following the patients for 3 years. Therefore, we can calculate which treatment was initially cheaper to provide, but more importantly, what happened in terms of cost relative to the survival of restorations and teeth for each arm.

Understanding how people recognize the impact of oral health on their lives has become an emergent topic in the scientific community. With regards to dental caries, most research has been published on the impact of the disease and its consequences and little on the consequences of providing curative care on the population’s quality of life. There have been some investigations of changes in OHRQoL associated with ART [37, 53]. However, to date, no research has been carried out to investigate the OHRQoL associated with the use of the HT.

Evidence from this study will expand the body of knowledge around ART and the HT, two minimally invasive treatments growing in popularity. The findings will also inform public health policy decisions as well as clinicians’, childrens’ and parent/carers’ choices for primary molar occluso-proximal caries lesions.

## Trial status

This trial is still currently recruiting participants.

## Additional files

Additional file 1: Case Report Form. (PDF 71 kb) Additional file 2: Questionnaires to be used in this study to evaluate the acceptance in relation to the treatments performed (children and parents version). These questionnaires are based on the questionnaires proposed by Bell et al. [31]. (DOCX 97 kb)

## Abbreviations

ART: Atraumatic Restorative Treatment
CONSORT: Consolidated Standards of Reporting Trials
CPQ: Child Perceptions Questionnaire
DMFT: decayed, missing, filled teeth in permanent teeth
dmft: decayed, missing, filled teeth in primary teeth
GIC: glass ionomer cement
HT: Hall Technique
ICER: incremental cost-effectiveness ratio
ITT: intention-to-treat
OHRQoL: oral health-related quality of life
OVD: occlusal-vertical dimension, P-CPQ, Parental-Caregiver Perceptions Questionnaire
PMC: preformed metal crown
WHO: World Health Organization
