# Supplementary material for: Atraumatic Restorative Treatment compared to the Hall Technique for occluso-proximal cavities in primary molars: study protocol for a randomized controlled trial
Source: Trials. 2016 Mar 31;17:169. doi: 10.1186/s13063-016-1270-z (PMC4815168; doi:10.1186/s13063-016-1270-z)
Supplement: Additional file 1: — Case Report Form. (PDF 71 kb) [file 13063_2016_1270_MOESM1_ESM.pdf]

Participant clinical record

**School:**		**Class:**
**Participant name:**		**Gender:**
**Birth date:** ___/___/___	**Municipality ID:**	
**Elastic:** ___/___/_____	**Wong-Baker:**<br>Initial =<br>Final =	**start:** ___:___<br>**finish:** ___:___

**1.**	**Participant ID:**<br>**(0-150)**		
	**Treatment date**	___/___/ _____	
**2.**	**Operator**		
	**Assistant**		
**3.**	**Treatment**	**ART** ☐	**HALL** ☐
**4.**	**Adjacent tooth**	**Mesial**: ( ) Present ( ) Absent	
		**Distal**: ( ) Present ( ) Absent	
	**Antagonist tooth**	( ) contact	( ) no contact
**5.**	**Tooth:**<br>________	**Cavitated surface:**<br>OM ☐ OD ☐	**DMFT/ dmft:**<br>__________
	**OC: ______mm**	**MD: ______mm**	**BL: ______mm**
**6.**	**OVD**	Initial =	Final =
		1 week =<br>1 month=<br>6 months=<br>12 months=<br>24 months=<br>36 months=	
**7.**	**Plaque index**	General =	
		Treated tooth =	
	**Gingival health**	General =	
		Treated tooth =	
**8.**	**Wong Baker faces scale**	Initial =	Final =
**9.**	**Time of treatments**	**start**<br>____: ____	**finish**<br>____: ____
