# Supplementary material for: Atraumatic Restorative Treatment compared to the Hall Technique for occluso-proximal cavities in primary molars: study protocol for a randomized controlled trial
Source: Trials. 2016 Mar 31;17:169. doi: 10.1186/s13063-016-1270-z (PMC4815168; doi:10.1186/s13063-016-1270-z)
Supplement: Additional file 2: — Questionnaires to be used in this study to evaluate the acceptance in relation to the treatments performed (children and parents version). These questionnaires are based on the questionnaires proposed by Bell et al. [31]. (DOCX 97 kb) [file 13063_2016_1270_MOESM2_ESM.docx]

| Questionário das crianças sobre a aceitação em relação aos tratamentos realizados | 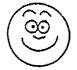  Concordo fortemente | 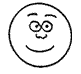  Concordo | 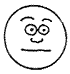  Indiferente | 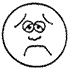  Discordo | 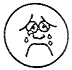  Discordo fortemente |
| --- | --- | --- | --- | --- | --- |
| 1. Você está feliz com seu dente que foi consertado? |  |  |  |  |  |
| 2. Você vai mostrar seu dente consertado aos seus amigos? |  |  |  |  |  |
| 3. Você achou que o dentista tratou você bem? |  |  |  |  |  |
| 4. Você entendeu tudo o que o dentista ia fazer? |  |  |  |  |  |
| 5. Você se incomodaria se as pessoas perguntassem e quisessem ver seu dente que foi consertado? |  |  |  |  |  |
| 6. Você está feliz com seu dente que foi consertado? |  |  |  |  |  |

| **Questionário dos pais sobre a aceitação em relação aos tratamentos realizados** | Concordo fortemente | Concordo | Indiferente | Discordo | Discordo fortemente |
| --- | --- | --- | --- | --- | --- |
| 1. Eu entendi o motivo de o meu filho necessitar de uma restauração. |  |  |  |  |  |
| 2. Não me incomoda a aparência da restauração/novo dente do meu filho. |  |  |  |  |  |
| 3. Eu acho que a restauração/novo dente do meu filho está realmente protegendo o dente do meu filho. |  |  |  |  |  |
| 4. Acredito que meu filho se sentiu bem durante o tratamento. |  |  |  |  |  |
| 5. Acredito que a equipe odontológica foi gentil e prestativa durante o tratamento do meu filho. |  |  |  |  |  |
